# Expression and functional study of DNA polymerases from *Psychrobacillus* sp. BL-248-WT-3 and FJAT-21963

**DOI:** 10.3389/fmicb.2024.1501020

**Published:** 2024-11-20

**Authors:** Yaping Sun, Danny Hsu Ko, Jie Gao, Kang Fu, Yuanchen Mao, Yun He, Hui Tian

**Affiliations:** Research Center of Molecular Diagnostics and Sequencing, Research Institute of Tsinghua University in Shenzhen, Shenzhen, China

**Keywords:** psychrophilic polymerases, protein expression, high ionic strength tolerance, strand displacement, exonuclease activity

## Abstract

The properties of DNA polymerases isolated from thermophilic and mesophilic microorganisms, such as the thermophilic *Geobacillus stearothermophilus* (Bst) and mesophilic *Bacillus subtilis phage* (Phi29), have been widely researched. However, DNA polymerases in psychrophilic microorganisms remain poorly understood. In this study, we present for the first time the expression and functional characterization of DNA polymerases PWT-WT and FWT-WT from *Psychrobacillus* sp. BL-248-WT-3 and FJAT-21963. Enzymatic activity assays revealed that FWT-WT possessed strand displacement but lacked exonuclease activity and high ionic strength tolerance, whereas PWT-WT lacked all these properties. Further protein engineering and biochemical analysis identified D423 and S490 as critical mutation sites for improving strand displacement and tolerance to high ionic strength, specifically in the presence of 0–0.3 M potassium chloride (KCl), sodium chloride (NaCl), and potassium acetate (KAc). Three-dimensional structural analysis demonstrated that the size and the electric charge of the single-stranded DNA (ssDNA) encapsulation entrance were pivotal factors in the binding of the ssDNA template.

## Introduction

Microorganisms in the environment survive across a range of temperatures and are categorized as thermophilic, mesophilic, and psychrophilic (D'Amico et al., 2006; Rabbani et al., 2023). DNA polymerases isolated from thermophilic and mesophilic microorganisms have been extensively studied, including well-characterized examples such as Phi29, Taq, T7, Klenow, and Bst polymerases (Kucera and Nichols, 2008; Ordonez and Redrejo-Rodriguez, 2023). DNA polymerases play a crucial role in DNA synthesis, ensuring faithful transmission of genetic information from one generation to another. These polymerases have three pivotal properties: strand displacement, tolerance to high ionic strength, and exonuclease activity; however, not all polymerases possess these features. Bst polymerases from the thermophilic *Geobacillus stearothermophilus* N3468 and Phi29 polymerase from the mesophilic *Bacillus subtilis phage* phi29 demonstrate significant strand displacement, facilitating downstream DNA movement during DNA synthesis. This property enables rolling circle amplification (RCA) and isothermal amplification (Kamtekar et al., 2004; Garafutdinov et al., 2020), eliminating the need for conventional denaturation, annealing, and extension stages and substantially contributes to molecular diagnostics, particularly for highly sensitive detection of bioanalytes (Garafutdinov et al., 2021). Notably, Phi29 polymerase is widely utilized in polymerase-nanopore sequencing systems (Stranges et al., 2016).

In addition to strand displacement, polymerases are sensitive to salt concentrations and their activities are inhibited at elevated salt levels (Zhai et al., 2019; Sun et al., 2023). Recent developments in ion current-based single-molecule nanopore sequencing technology necessitate DNA polymerases synthesizing DNA under high-salinity conditions, which is crucial for optimal nucleobase recognition (Fuller et al., 2016). Although thermophilic halophilic polymerases from the Red Sea have been reported to work in high salt concentration condition (Takahashi et al., 2018), the existence of psychrophilic polymerases with high ionic strength tolerance remains unknown.

Besides strand displacement and high ionic strength tolerance, the intrinsic 3′ – 5′ exonuclease activity of DNA polymerases plays a primary role in proofreading polymerization errors and maintaining genetic stability (Betancurt-Anzola et al., 2023). Phi29, T7, Klenow, and Taq polymerases are endowed with an intrinsic 3′ – 5′ exonuclease activity, removing nucleotides at the 3′ end, while Bst polymerase lacks exonuclease activity (Aliotta et al., 1996; Del Prado et al., 2019; Green and Sambrook, 2020; Sun et al., 2023). However, high exonuclease activity has impeded the widespread application of polymerases in polymerase-nanopore sequencing systems due to the degradation of oligonucleotide-modified substrates.

DNA polymerases in psychrophilic microorganisms is yet to be reported. The PIPI-WT polymerase from *Psychromonas ingrahamii* has been shown to perform RCA at 30°C – 40°C (Xue et al., 2021), though its compatibility with other critical properties remains elusive. This study presents, for the first time, the protein expression, strand displacement, exonuclease activity, and ionic strength tolerance of the DNA polymerases PWT-WT and FWT-WT from *Psychrobacillus* sp. BL-248-WT-3 and FJAT-21963. Through structural simulation, protein engineering, and biochemical analysis, D423 and S490 in PWT-WT and FWT-WT were identified as critical sites for enhancing strand displacement and high ionic strength tolerance. This study provides valuable insights into the isolation and characterization of DNA polymerases from psychrophilic microorganisms.

## Materials and methods

### Plasmid construction and protein expression

The DNA sequences of Phi29, PWT-WT, FWT-WT, PIPI-WT, FWT-MT, PWT-MT, FWT-D423A_S490R, FWT-D423A_S490A, FWT-D423A_F491A, FWT-S490A_F491A, and FWT-S490R_F491A with TEV cleavable His-tags at the C-terminus, were optimized toward *Escherichia coli* (*E. coli*) and synthesized by Genscript Biotechnology (Nanjing, China). These sequences were cloned into *Xba*I and *Xho*I sites of the pET21a plasmid. Restriction-endonucleases *Xba*I, *Xho*I and T4 DNA ligase were used for digestion and ligation (Genscript Biotechnology, Nanjing, China). All expression plasmids were transformed into BL21 competent *E. coli* cells according to the instructions provided by the manufacturer (TOLOBIO, Wuxi, China). The detailed protocol for the plasmid transformation is provided in the Supplementary materials. Three colonies for each protein were cultured in 5 mL lysogeny broth (LB) medium supplemented with ampicillin (50 μg/mL) overnight at 37°C. Two expression conditions were tested. For the first assay, the cells were incubated on a shaker (200 rpm) in 200 mL of liquid LB medium at 37°C until an OD_600_ value of 0.6 was achieved. Isopropyl *β*-D-thiogalactopyranoside (IPTG, Sango, China) was added at a final concentration of 1.2 mM to induce protein expression. The incubation temperature was lowered from 37°C to 16°C, and protein expression was carried out for 14 h – 16 h. For the second assay, the cells were incubated at 37°C until an OD_600_ value reached 1.5–1.6. The final concentration of IPTG was 1.2 mM. The incubation temperature was lowered from 37°C to 20°C, and protein expression was carried out for 24 h. After incubation, the cells were centrifuged at 8000 rpm for 10 min, collected, and stored at −80°C until further use.

His-tagged target proteins were purified using an in-house Ni Sepharose 6FF gravity column at 4°C. The details of purification using the Ni Sepharose column are provided in the Supplementary materials. Polymerase proteins were eluted using elution buffers [50 mM Tris–HCl (pH 7.5) and 500 mM NaCl with different concentrations of imidazole]. Target proteins were detected via in-house 10% sodium dodecyl sulfate-polyacrylamide gel electrophoresis (SDS-PAGE). High-purity elution components were concentrated using 10 K ultrafiltration [157,655 (24), Millipore] to remove imidazole and increase protein concentration. To remove His-tags, 400 μL of *tobacco etch virus* (TEV) protease (Beyotime, Shanghai, China) were used to digest 600 μg of target protein overnight at 4°C in TEV buffer (50 mM Tris–HCl, 5 mM NaCl, 1 mM EDTA, 1 mM DTT, pH 8.0). The His-tagged TEV protease and cleaved His-tags were removed by a Ni Sepharose 6 Fast Flow column, and target proteins were collected in the flow-through. Purification was confirmed by 10% SDS-PAGE and subjected to 10 K ultrafiltration to obtain highly concentrated protein solutions. Protein concentrations were calculated using a Pierce BCA Protein Assay Kit (Thermo Fisher Scientific Inc., Cleveland, OH, United States). Protein purity was tested by in-house SDS-PAGE. Protein concentrations were checked using bovine serum albumin (BSA) as the reference standard. The details of in-house SDS-PAGE are provided in the Supplementary materials.

### Structural analysis

Polymerase structures were generated using the AlphaFold2 Colab notebook (Cramer, 2021; Van Breugel et al., 2022). The Klenow (1KLN) polymerase structure was downloaded from the Protein Data Bank (Burley et al., 2022). The structure analysis and electrostatic potential maps of the surface were created and analyzed using PyMOL (Kagami et al., 2020).

### Exonuclease activity

The exonuclease activity of DNA polymerases was tested using a specific M13mp18 single-stranded DNA (ssDNA) primer M13pd (5’-CGCCAGGGTTTTCCCAGTCACGAC-3′) under two experimental conditions. Phi29 DNA polymerase was used as a positive control because of its strong exonuclease activity. Briefly, the first condition involved mixing 200 nM polymerases with 0.5 μM M13pd in a 1 × phi29 Pol reaction buffer (New England BioLabs, Cambridge, United Kingdom) and adjusting the final volume to 20 μL. The second condition involved mixing 200 nM polymerases with 1 μM M13pd and 300 mM KCl in a 1 × phi29 Pol reaction buffer and adjusting the final volume to 20 μL. The reactions were performed at 30°C for 10, 60, and 180 min. The results were visualized using 10% TBE-urea gel in an XCell SureLock Mini-Cell (Thermo Fisher Scientific Inc., Cleveland, OH, United States), as instructed by the manufacturer. All gel electrophoresis were performed at 180 V for 45 min and stained with SYBR Gold (Invitrogen, Carlsbad, CA, United States). O’RangeRuler 10 bp DNA Ladder (Thermo Fisher Scientific Inc., Cleveland, OH, United States) was used as a DNA molecular weight marker.

### Rolling circle amplification

The template-primer complex was prepared by mixing 1 μM of M13pd with 200 nM of M13mp18 ssDNA (New England BioLabs, Cambridge, United Kingdom). The mixture was heated at 95°C for 3 min in a 1 × phi29 Pol reaction buffer. The complexes were incubated at 25°C for 25 min.

To explore processivity and ionic strength tolerance, 20 nM of the template-primer complex, comprising 20 nM template and 100 nM primer, was mixed with 200 nM of polymerase proteins under various salt concentrations: no extra salt, 150 mM KCl, 300 mM KCl, 150 mM NaCl, 300 mM NaCl, 150 mM KAc, and 300 mM KAc. The reactions were performed at 30°C for 180 min, and then terminated by adding 0.5 M EDTA. Each assay was repeated thrice for each polymerase.

To identify the key mutant sites, 200 nM of each of the FWT mutant variants (FWT-D423A_S490R, FWT-D423A_S490A, FWT-D423A_F491A, FWT-S490A_F491A, and FWT-S490R_F491A) was mixed with 20 nM template-primer complex to react at 30°C for 180 min under 150 mM KCl and 300 mM KCl conditions. Each assay was repeated three times.

RCA products were detected using 0.6% alkaline agarose gel electrophoresis. GeneRuler High Range DNA Ladder (Thermo Fisher Scientific Inc., Cleveland, OH, United States) and 1 Kb Plus DNA Ladder (TransGene, Beijing, China) were used as DNA molecular weight markers. A peristaltic pump (BT100-1 L; LongerPump Co., Ltd., Baoding, China) circulated the running buffer (46 mM NaCl, 1 mM EDTA, 4 mM NaOH, pH 10) maintain gel temperature. After 2–3 h, the gel was stained with SYBR Gold for 30 min, and the intensity and length of the RCA products were analyzed using AzureSpot.

## Results

### Protein expression analysis of psychrophilic polymerases

**Figure 1:**
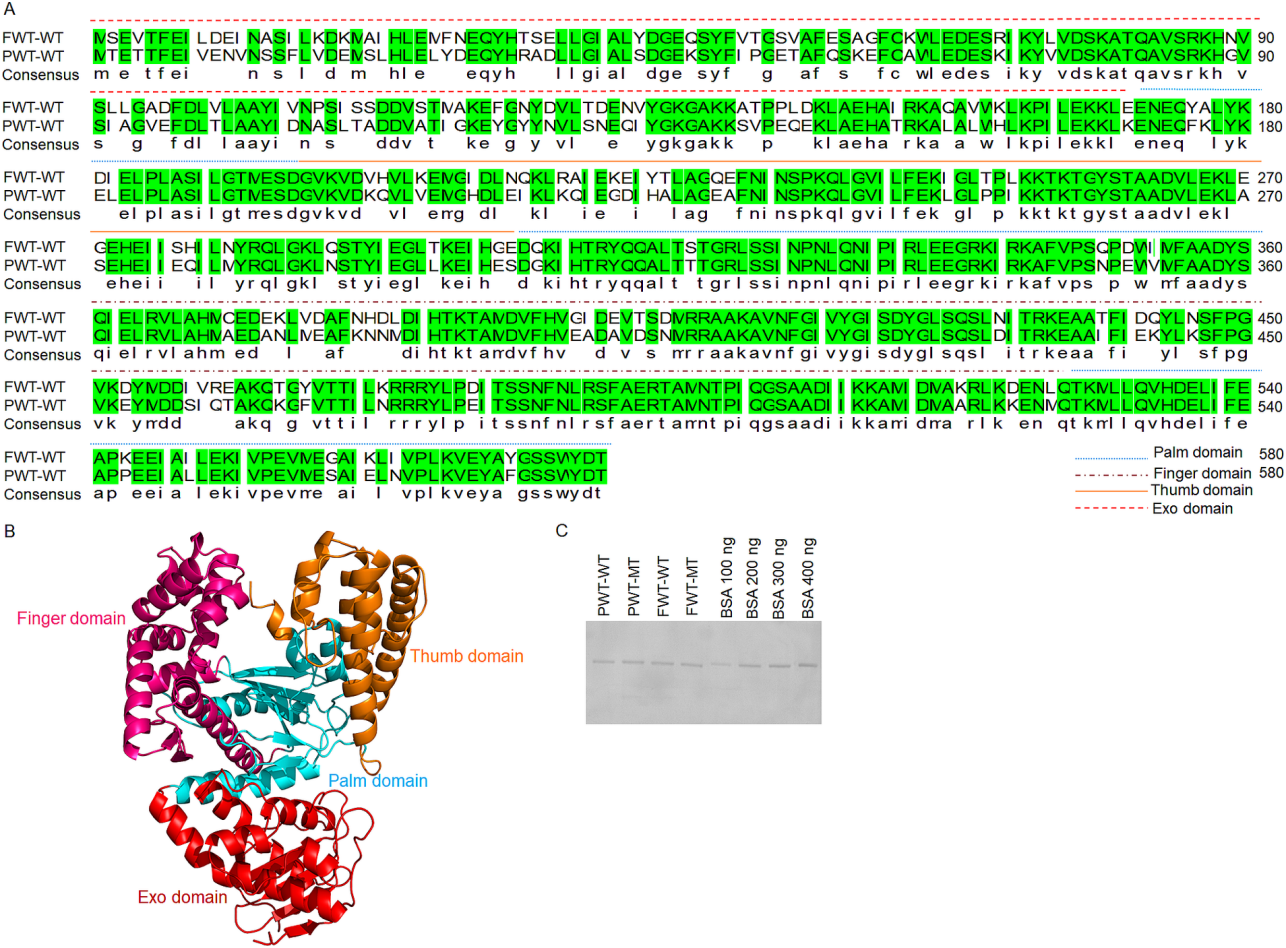
Sequence alignments and structural analysis of PWT-WT and FWT-WT polymerases. **(A)** Polymerase sequence alignments of PWT-WT and FWT-WT. Different line styles represent the palm, finger, thumb, and exonuclease domains of the polymerases. Green indicates identical amino acids in both FWT-WT and PWT-WT. **(B)** Functional domain analysis of family A polymerases. Red represents the exonuclease domain, pink represents the finger domain, orange represents the thumb domain, and blue represents the palm domain. **(C)** Protein testing for PWT-WT, FWT-WT, PWT-MT and FWT-MT. After calculating protein concentrations using the Pierce BCA Protein Assay Kit, 300 ng of PWT-WT, FWT-WT, PWT-MT and FWT-MT polymerase proteins were further checked with reference BSA standards in the 10% SDS-PAGE.

Protein sequences were obtained from the National Center for Biotechnology Information (NCBI) database to screen for potential psychrophilic polymerase candidates. PWT-WT (accession no. WP_169360306.1) and FWT-WT (accession no. WP_056833027.1) were selected as two additional family A polymerase candidates, derived from *Psychrobacillus* sp. BL-248-WT-3 and FJAT-21963, respectively. This study provides the first comprehensive analysis of protein expression, protein purification, exonuclease activity, strand-displacement, and processivity of the selected polymerases, aiming at exploring their functional regulatory mechanisms to enhance polymerase activities. The sequences are summarized in Figure 1A. Figure 1B shows the functional domains of the family A psychrophilic polymerases. The palm domain serves as the active center, in which the transfer of phosphoryl groups occurs. The thumb domain stabilizes the template-primer complex. The finger domain plays a role in the processivity and strand displacement. The exonuclease domain is essential for removing incorrect nucleotides to maintain the high fidelity of polymerases.

Initial efforts to obtain highly purified psychrophilic polymerase proteins involved attempting traditional prokaryotic expression conditions (OD = 0.6, 16°C for 14–16 h in *E. coli* BL21). However, protein yields were low. We further optimized the experimental conditions by considering polymerases from psychrophilic microorganisms. When the OD reached 1.5–1.6, proteins were expressed at 20°C for 24 h. Purification was achieved *in vitro* using His-tags, and the enough proteins were used for subsequent polymerase activity testing. Protein concentration and purity were determined using Pierce BCA Protein Assay Kit and SDS-PAGE (Figure 1C). The protein concentration was maintained at less than 600 ng/μL, as higher concentration easily resulted in precipitation.

### Exonuclease activity analysis of psychrophilic polymerases

**Figure 2:**
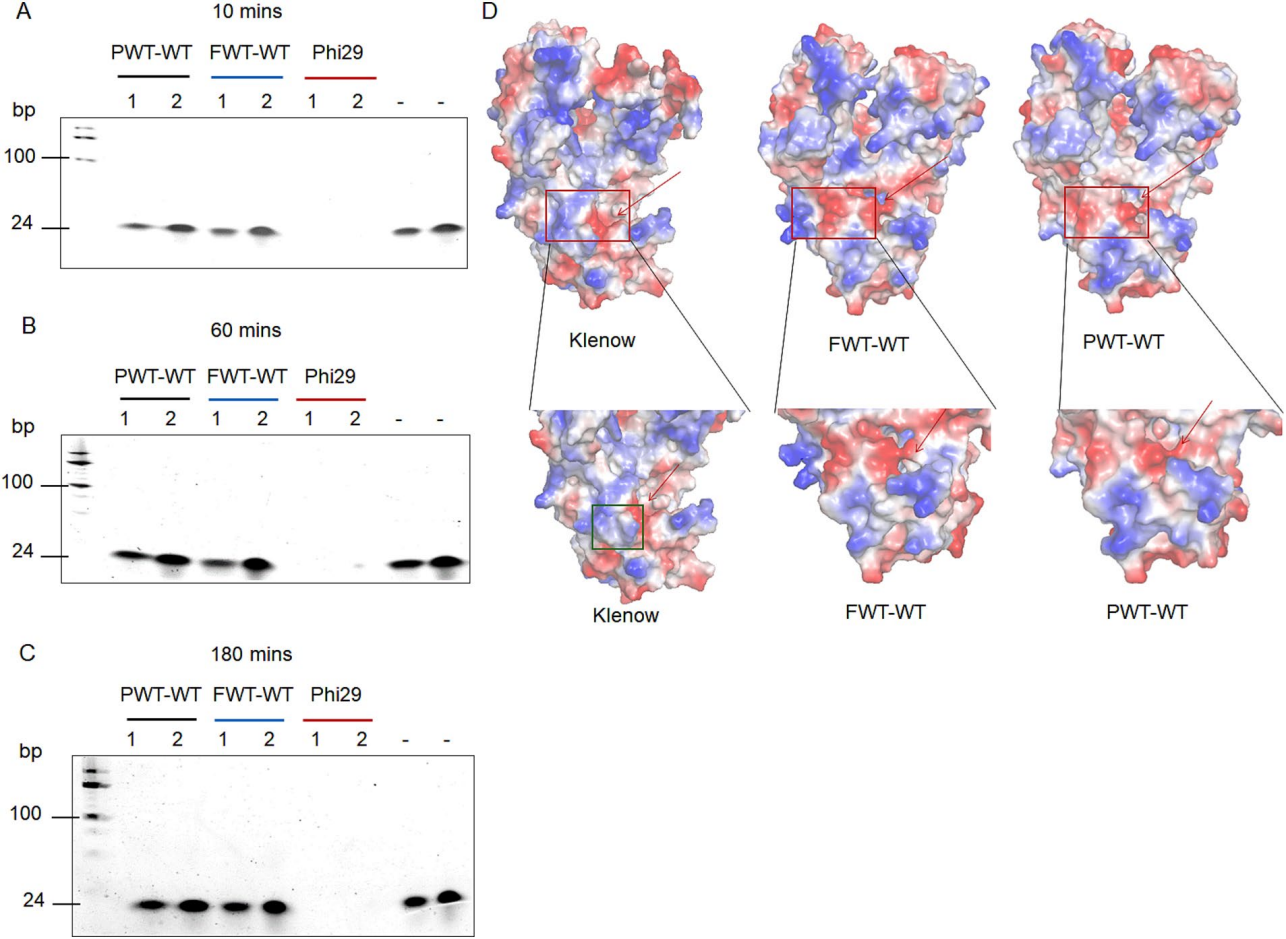
3′ – 5′exonuclease activity analysis of PWT-WT, FWT-WT and Phi29 polymerases. **(A–C)** Exonuclease activity assay under different salt concentration conditions and time points. Lane 1 refers to 200 nM polymerase and 0.5 μM oligonucleotides (M13pd) in 1 × phi29 Pol reaction buffer; Lane 2 refers to 200 nM polymerase, 1 μM oligonucleotides (M13pd), and 300 mM KCl in 1 × phi29 Pol reaction buffer; “-” denotes conditions with 0.5 μM and 1 μM oligonucleotides (M13pd), respectively. **(D)** Charged surface analysis of Klenow, FWT-WT, and PWT-WT polymerases using PyMOL. The red arrow and green square indicate the exonuclease active pocket and the specific protrusion domain, respectively.

To explore the exonuclease activities of the two psychrophilic polymerases, we compared them by incubating 200 nM of each polymerase with 0.5 μM or 1 μM oligonucleotides (M13pd) under varying time points and salt concentrations. In the salt-free group, 0.5 μM of oligonucleotides were added, while in the high salt group, 1 μM were utilized. Phi29 polymerase (accession no. P03680) has been reported to possess strong strand displacement and high exonuclease activity (Zhou et al., 2024). We used Phi29 polymerase as a positive control. Based on the TBE gel analysis, PWT-WT and FWT-WT exhibited no discernible exonuclease activity under varying time and salt concentration conditions compared to the Phi29 polymerase (Figures 2A–C).

To further investigate the regulatory mechanisms of exonuclease activity in PWT-WT and FWT-WT polymerases, we predicted protein structures using AlphaFold2 and analyzed them with PyMOL (Cramer, 2021). Structural comparisons with Klenow revealed a basic structural protrusion around the exonuclease active pocket as a potential regulator of exonuclease activity. As shown in Figure 2D, Klenow polymerase with a specific structure (green square) showed robust exonuclease activity (Kukreti et al., 2008), whereas PWT-WT and FWT-WT polymerases lacked the described structural features, resulting in their failure to degrade oligonucleotides.

### Enhancing strand displacement and high ionic tolerance in psychrophilic polymerases

**Figure 3:**
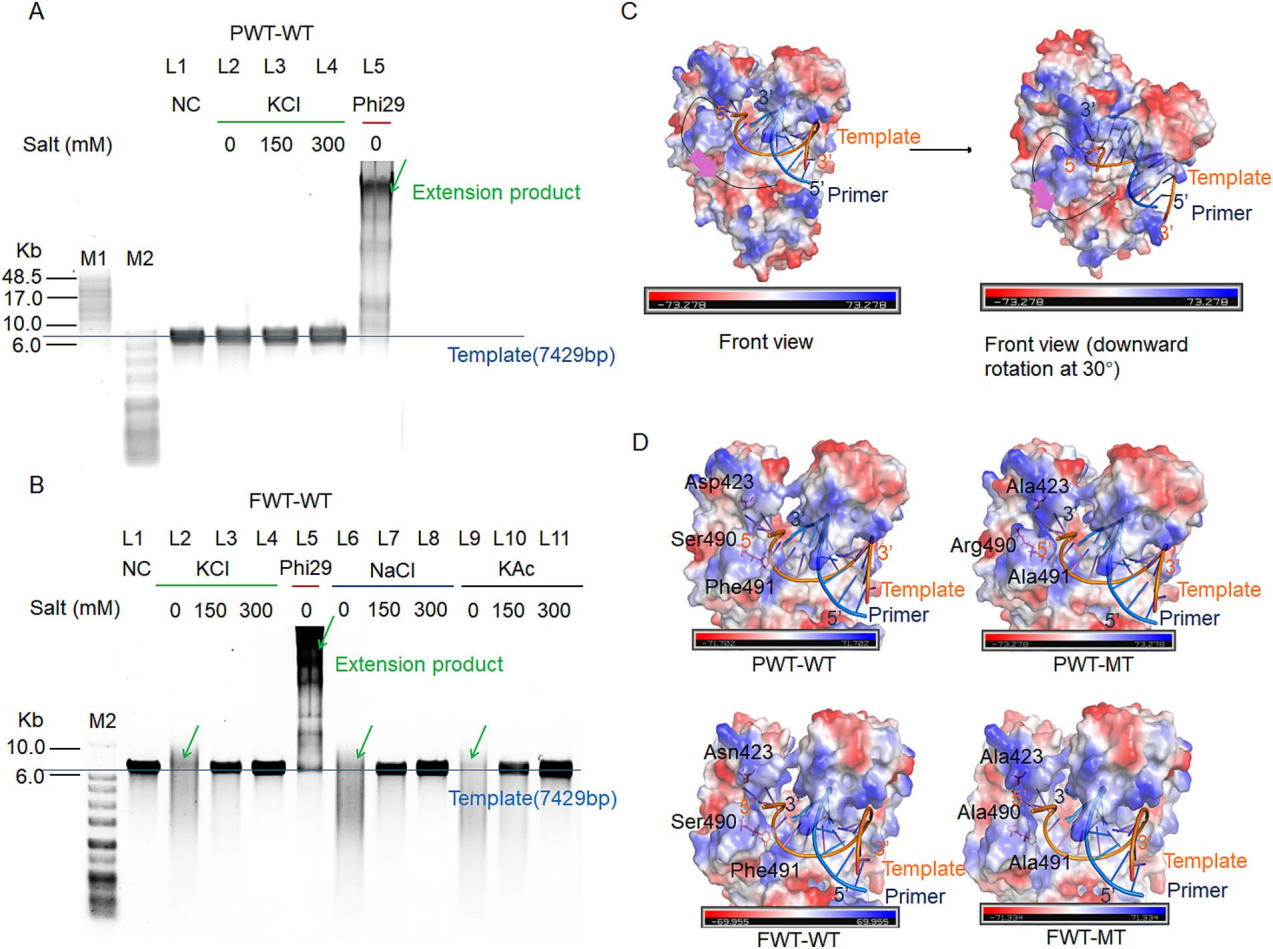
RCA efficiency and ionic strength tolerance ability of PWT-WT and FWT-WT polymerases. **(A,B)** RCA assays for PWT-WT **(A)** and FWT-WT **(B)**. The replication reactions with 200 nM polymerases and a 20 nM template-primer complex, run at 30°C for 3 h under various salt ion and concentration conditions (no extra salt, 150 mM KCl, 300 mM KCl, 150 mM NaCl, 300 mM NaCl, 150 mM KAc, and 300 mM KAc). NC refers to the negative control with only the M13mp18 DNA template. Phi29 polymerase serves as a positive control under on-salt condition. Green arrows indicate the extension product; blue line indicates the position of the template. M1 refers to the GeneRuler High Range DNA Ladder; M2 refers to the 1 KB Plus DNA Ladder. **(C)** Strand displacement process model. The black lines indicate the track of single-stranded DNA, while purple arrows indicate the direction of the template strand entering the exonuclease pocket for strand displacement facilitation. **(D)** Surface charge and mutant site analysis of PWT-WT, PWT-MT, FWT-WT, and FWT-MT. The blue line represents the primer strand, and the orange line represents the template strand.

FWT-WT and PWT-WT were identified as potential new polymerases using the NCBI database. Notably, both FWT-WT and PWT-WT exhibited negligible exonuclease activity (Figure 2). To assess their ionic strength tolerance and strand displacement properties, we conducted RCA using various salt concentrations (salt-free, 150 mM KCl, 300 mM KCl, 150 mM NaCl, 300 mM NaCl, 150 mM KAc, and 300 mM KAc) with M13mp18 ssDNA as a template. Phi29 polymerase, serving as a positive control, showed strong extension ability under salt-free condition, indicating that polymerases with strand displacement could perform RCA in the initial buffer condition. As depicted in Figures 3A,B, PWT-WT demonstrated poor performance in isothermal amplification owing to the lack of strand-displacement activity, whereas FWT-WT showed otherwise. However, high concentrations of KCl, NaCl, and KAc significantly inhibited the extension activity of FWT-WT, even though it could perform RCA under salt-free conditions (Figure 3B). In response, we developed a model for the strand displacement process and embarked on engineering these polymerases to enhance their strand displacement and high ionic strength tolerance properties through structural analysis in PyMOL. The model accounted for the 3′ terminus of the ssDNA template, which entered the semi-circular pocket to anneal with the 5′ terminus of a primer. Subsequently, the 5′ terminus of the template proceeded into the exonuclease domain for strand displacement (illustrated with purple arrow in Figure 3C). The size and electric charge of the semi-circular pocket are important factors in the binding of a ssDNA template. We identified this specific semi-circular structure as the “ssDNA encapsulation entrance.”

**Figure 4:**
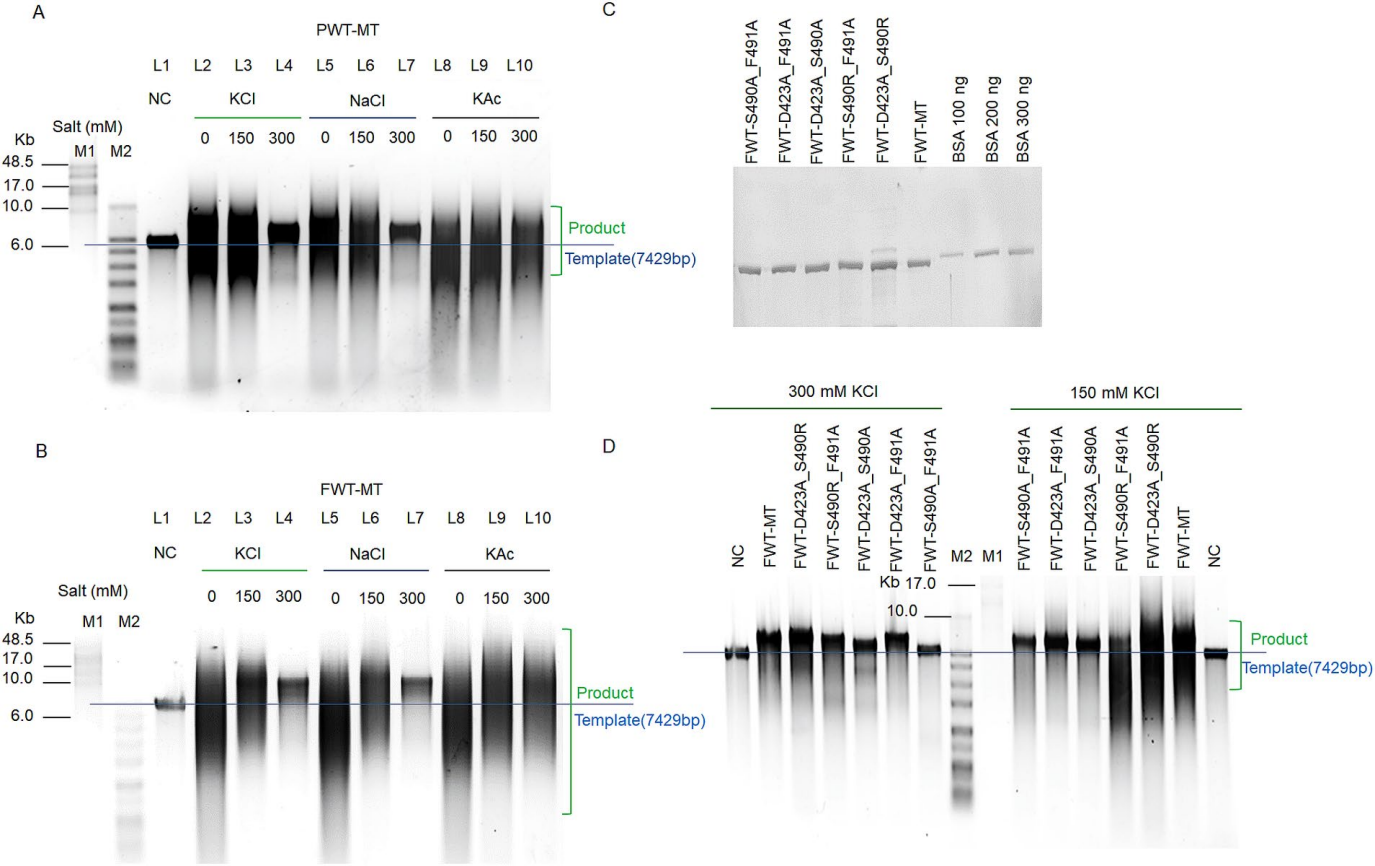
Enhanced RCA efficiency and ionic strength tolerance of polymerase mutant variants. **(A,B)** RCA assays of PWT-MT (A) and FWT-MT **(B)** polymerases using M13mp18 DNA as templates under different salt concentration conditions. Replication with 200 nM polymerases and a 20 nM template-primer complex was performed at 30°C for 3 h under various salt and concentration conditions (no extra salt, 150 mM KCl, 300 mM KCl, 150 mM NaCl, 300 mM NaCl, 150 mM KAc, and 300 mM KAc). NC refers to the negative control with only the template. **(C)** Protein testing of FWT-D423A_S490R, FWT-D423A_S490A, FWT-D423A_F491A, FWT-S490A_F491A, and FWT-S490R_F491A. After calculating purified protein concentrations by the Pierce BCA Protein Assay Kit, 800 ng of samples were compared to the BSA standards in a 10% SDS-PAGE. **(D)** RCA assays for FWT-D423A_S490R, FWT-D423A_S490A, FWT-D423A_F491A, FWT-S490A_F491A, and FWT-S490R_F491A polymerases. For these assays, 200 nM polymerases were mixed with a 20 nM template-primer complex, consisting of 20 nM template and 100 nM primer, and extended at 30°C for 3 h under different salt concentration conditions (150 mM KCl and 300 mM KCl). M1 refers to the GeneRuler High Range DNA Ladder; M2 refers to the 1 KB Plus DNA Ladder. Green box denotes the extension product; Blue line indicates the position of the template.

Based on the above hypothesis, to endow strand displacement with PWT-WT, we mutated D423, Ser490, and Phe491 to Ala, Arg, and Ala, respectively, resulting in the PWT-Mutant-type (PWT-MT). D423A and S490R reduced the acidic charge and increased alkaline charge of “ssDNA encapsulation entrance,” further enhancing template binding, while F491A reduced the steric hindrance of amino acid side chain (Figure 3D). The three mutant sites efficiently elevated the strand-displacement activity of PWT-MT, which was purified by His-tags, enabling it to perform RCA under different salt concentrations (Figure 4A).

To further explore the effects of these mutant sites on strand displacement, we mutated D423, S490, and F491 in FWT-WT to Ala, Ala, and Ala, resulting in the FWT-mutant type (FWT-MT). Unlike S490R in PWT-MT, S490A in FWT-MT was designed to investigate whether increasing the alkaline charge was necessary to improve stand displacement (Figure 3D). FWT-MT purified by His -tags exhibited enhanced processivity compared to FWT-WT at high ionic concentrations (Figure 4B).

Interestingly, in addition to the improvement in strand displacement, PWT-MT and FWT-MT exhibited significantly higher ionic strength tolerance property when subjected to high concentrations of KCl, NaCl, and KAc (Figures 4A,4B). However, their processivity decreased with increasing salt concentration. Both FWT-MT and PWT-MT were observed to produce longer and more dispersive products at 300 mM KAc than that at 300 mM KCl and 300 mM NaCl (Figures 4A,B).

We also tested the ionic strength tolerance and processivity of PIPI-WT polymerase (accession no. WP_011771427.1), derived from the psychrophilic bacterium *Psychromonas ingrahamii.* PWT-WT could not perform amplification because of the lack of strand displacement, serving as a negative control (Supplementary Figure S1). Under the salt-free condition, RCA products from PIPI-WT polymerase showed wide-ranging sizes. When 150 mM KCl was added, RCA products centralized into longer fragments. This phenotype was particularly pronounced under 300 mM KCl (Supplementary Figure S1). Importantly, PIPI-WT polymerase exhibited processivity similar to that of PWT-MT and FWT-MT under high salt conditions.

### D423A and S490R as key mutant sites to regulate strand displacement and ionic strength tolerance of psychrophilic polymerase

To further explore the key mutant sites contributing to strand displacement and ionic strength tolerance, we investigated the effects of double amino acid replacement in FWT-MT. Five mutant variants were constructed as follows: FWT-D423A_S490R, FWT-D423A_S490A, FWT-D423A_F491A, FWT-S490A_F491A, and FWT-S490R_F491A. The polymerase proteins were purified using His-tags (Supplementary Figure S2). Protein concentrations were measured using the Pierce BCA Protein Assay Kit and validated against BSA standards (Figure 4C).

Based on the RCA results under 150 mM and 300 mM KCl conditions, FWT-D423A_F491A and FWT-S490R_F491A exhibited similar activity to FWT-MT (D423A, S490A, and F491A) under 300 mM KCl conditions, whereas FWT-S490A_F491A showed the weakest extension ability under both conditions (Figure 4D). Comparison between FWT-D423A_F491A and FWT-D423A_S490A indicated that F491A improved higher polymerase activity than S490A in 300 mM KCl (Figure 4D). Moreover, FWT-D423A_S490R showed higher polymerase activity than FWT-D423A_F491A, suggesting that the effect of S490R on polymerase activity was more critical than that of F491A. Interestingly, FWT-D423A_S490R demonstrated enhanced polymerase activity compared to that of FWT-MT.

## Discussion

Extensive studies have documented the activities of DNA polymerases isolated from thermophilic and mesophilic microorganisms. Exonuclease activity, ionic strength tolerance and strand displacement are crucial properties of polymerases for their application as diagnostic tools, particularly in polymerase-nanopore sequencing systems (Fuller et al., 2016; Palla et al., 2021; Oscorbin and Filipenko, 2023). However, few studies have been conducted on psychrophilic polymerases. In this study, we introduced the expression and functional properties of two distinctive psychrophilic polymerases, PWT-WT and FWT-WT, from the family A polymerases. Structural, bioengineering, and biochemical analysis further identified D423 and S490 as key active sites to regulate strand displacement and high ionic strength tolerance in psychrophilic polymerases.

Proofreading during DNA synthesis requires the exonuclease activity of polymerases (Bebenek and Ziuzia-Graczyk, 2018; Czernecki et al., 2023). But strong exonuclease activity could degrade the oligonucleotide-modified substrates, further disturbing the detection of sequencing signals in polymerase-nanopore sequencing system (Fuller et al., 2016; Sun et al., 2024). Known Phi29 polymerase has been modified to remove the exonuclease activity for compatibility with next-generation sequencing systems (Sun et al., 2023). Bst polymerase from *Geobacillus stearothermophilus* does not possess 3′ – 5′ exonuclease activity, but its higher reaction temperature (60–65°C) limited its application in sequencing technology (Oscorbin and Filipenko, 2023). The psychrophilic polymerases PWT-WT and FWT-WT are members of family A polymerases without exonuclease activity, presenting as potential polymerase candidates for next-generation sequencing application.

**Figure 5:**
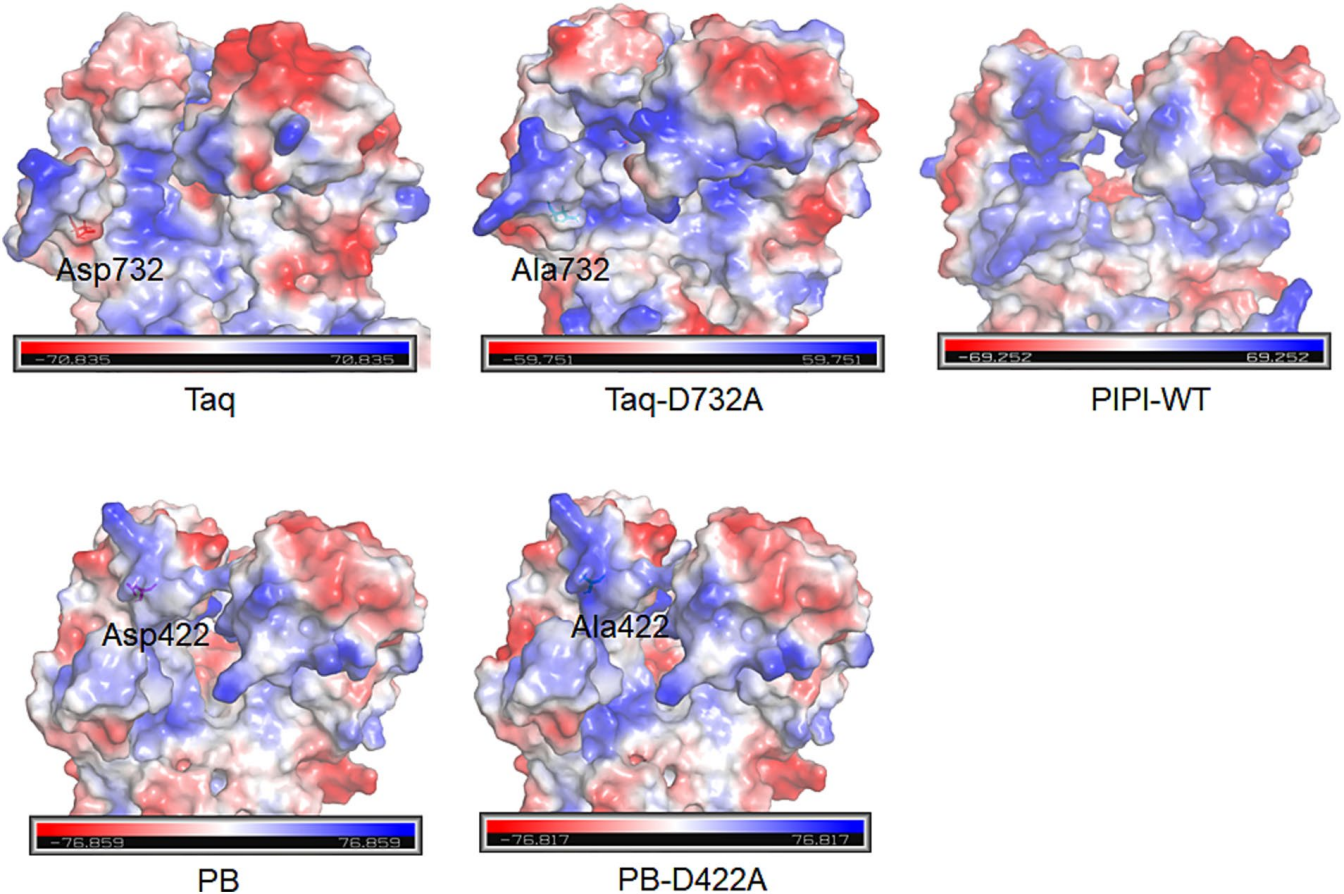
Charged surface analysis of single-stranded DNA encapsulation entrances for PB, Taq and PIPI-WT polymerases using PyMOL. D422A in PB polymerase and D732A in Taq polymerase are shown.

Strand displacement is defined as the amplification capability of polymerases using ssDNA, facilitating RCA and multiple displacement amplification for whole-genome amplification technique (Ordonez and Redrejo-Rodriguez, 2023). Although PWT-WT is unable to perform RCA, PWT-MT successfully demonstrated this ability. PIPI-WT polymerase, derived from the psychrophilic bacterium *Psychromonas ingrahamii* has been reported to possess DNA extension activity (Xue et al., 2021). The RCA results for PWT-MT under high salt conditions were similar to those of PIPI-WT polymerase. A possible reason for this is that the D423A/S490R/F491A mutation in PWT-MT increased the alkaline charge of its ssDNA encapsulation entrance, thus enhancing its strand displacement. The D422A mutant has been reported to increase the strand displacement activity of PB polymerase from *Psychrobacillus* sp. (Piotrowski et al., 2019). D732N mutation endowed Taq DNA polymerase from *Thermus aquaticus* with strand displacement activity (Barnes et al., 2020). Their structural analysis revealed the improvement in the size and electric charge of single-stranded DNA encapsulation entrance (Figure 5). This supports our hypothesis on enhancing strand displacement activity is suitable not only for the psychrophilic polymerases, PWT-WT and FWT-WT, but also may be applicable to other polymerases, warranting further investigation. Expanding this approach could broaden the application of diverse polymerases, such as in single-molecule sequencing systems.

A high ionic strength tolerance enables polymerases to function at high salt concentrations (Wang et al., 2004; Zhai et al., 2019; Sun et al., 2023; Sun et al., 2024). The helix-hairpin-helix (HhH)_2_ domain of *Methanopyrus kandleri* topoisomerase V has been successfully fused with Taq, Pfu and Phi29 DNA polymerases to increase high ionic strength tolerance (Pavlov et al., 2002; Gao et al., 2021). The exonuclease domain of PB polymerases from *Psychrobacillus* sp. were replaced with that of Bst polymerases to remove the exonuclease activity and enhance the high ionic strength tolerance (Sun et al., 2024). The imbalance between acidic and basic amino acids is a prime factor for tailoring high ionic strength tolerance. Increasing the number of acidic amino acids on the surface reportedly promotes halotolerance in carbonic anhydrases (Warden et al., 2015) and contributes to halophilic choline kinase activity (Zheng et al., 2019). Analysis of the ratio of acidic-to-basic amino acids in PWT-WT and FWT-WT polymerases revealed a higher proportion of acidic residues in both (Supplementary Table S1). However, neither polymerases were effective under high ionic strength conditions, but D423A/S490A or S490R /F491A mutations improved the ionic strength tolerance in PWT-MT and FWT-MT polymerases by reducing the hindrance at the ssDNA encapsulation entrance. The size and electric charge of the ssDNA encapsulation entrance have emerged as vital structural features governing the polymerase strand displacement capabilities. Strand displacement enhancement is another method for improving ionic strength tolerance.

Interestingly, through testing the polymerase activity of PWT-MT and FWT-MT under various salt types and concentrations, chloride ion was found to have a stronger negative effect on polymerase activity than acetate ion. Phi29 and VPV262 polymerases have been reported to possess higher ionic strength tolerance under KAc conditions than that under KCl conditions (Gao et al., 2020; Gao et al., 2021). Klenow and Klentaq polymerases demonstrated stronger binding affinities in glutamate buffers than in chloride buffers (Deredge et al., 2010), suggesting that the ion choice influences polymerase activity. This insight may assist with buffer selection to improve polymerase performance and signal generation in future polymerase-nanopore sequencing systems.

## Conclusion

We successfully expressed, purified and characterized the DNA polymerases PWT-WT and FWT-WT from *Psychrobacillus* sp. BL-248-WT-3 and FJAT-21963. Our findings indicated that these enzymes belonged to the family A DNA polymerase, featuring four conserved domains and lacking exonuclease activity. Importantly, we identified D423 and S490 as crucial mutant sites for enhancing strand displacement and ionic strength tolerance, particularly in the presence of 0–0.3 M KCl, NaCl and KAc. By elucidating these properties, our study provides a valuable reference for the isolation and characterization of DNA polymerases from psychrophilic microorganisms. Future research should explore the regulatory mechanisms of psychrophilic DNA polymerases for potential applications in various fields.

## Data Availability

The original contributions presented in the study are included in the article/Supplementary material, further inquiries can be directed to the corresponding authors.
