# Supplementary material for: Expression and functional study of DNA polymerases from Psychrobacillus sp. BL-248-WT-3 and FJAT-21963
Source: Front Microbiol. 2024 Nov 20;15:1501020. doi: 10.3389/fmicb.2024.1501020 (PMC11615080; doi:10.3389/fmicb.2024.1501020)
Supplement: Supplementary file 1 [file Table_1.docx]

**Supplementary Table 1** Ratio of acidic and basic amino acids in PIPI-WT, PWT-WT, and FWT-WT polymerases.

| Gene name | PI | Acidic / Basic amino acids |
| --- | --- | --- |
| PIPI-WT | 5.57 | (Asp + Glu) : (Arg + Lys) = 83: 70 |
| PWT-WT | 5.41 | (Asp + Glu) : (Arg + Lys) = 87: 69 |
| FW-WT | 5.43 | (Asp + Glu) : (Arg + Lys) = 87: 70 |
