# Supplementary material for: Expression and functional study of DNA polymerases from Psychrobacillus sp. BL-248-WT-3 and FJAT-21963
Source: Front Microbiol. 2024 Nov 20;15:1501020. doi: 10.3389/fmicb.2024.1501020 (PMC11615080; doi:10.3389/fmicb.2024.1501020)
Supplement: Supplementary file 2 [file Data_Sheet_1.docx]

Supplementary Material

Expression and functional study of DNA polymerases from Psychrobacillus sp. BL-248-WT-3 and FJAT-21963

Yaping Sun^1^, Danny Hsu Ko^1^, Jie Gao^1^, Kang Fu^1^, Yuanchen Mao^1^, Yun He^1,^* and Hui Tian^1,^*

^1^ Research Center of Molecular Diagnostics and Sequencing, Research Institute of Tsinghua University in Shenzhen, Shenzhen, 518000, China

*** Correspondence:**

Corresponding Author: [hej@tsinghua-sz.org](mailto:hej@tsinghua-sz.org); [tianhui@tsinghua-sz.org](mailto:tianhui@tsinghua-sz.org)

# 1 Plasmid transformation

BL21 competent *E. coli* cells were placed on ice for 2 min. Next, 100 ng of plasmid was added, and the mixture was immediately placed on ice for an additional 30 min. After the ice bath, the mixture was incubated in a 42°C metal bath (H203-100C, COYOTE, China) for 90 sec, and then placed on ice for 3 min. 500 μL LB medium without antibiotic was added to the mixture to culture on a shaker (200 rpm) at 37°C for 45 min. After centrifugation, the cells were resuspended in 200 μL LB medium without antibiotic and were cultured on a 10 cm LB agar plate containing ampicillin overnight.

# In-house SDS-PAGE

# In-house SDS-PAGE were prepared using a 10% PAGE gel quick preparation kit (Epizyme, Shanghai, China). For protein electrophoresis, 10 μL of protein samples and 2.5 μL of 6 × protein loading buffer (TransGene, Beijing, China) were mixed, then heated at 95℃ for 5 min and centrifuged at 12000 rpm for 1 min. The tris-glycine buffer was used for electrophoresis, which was performed at 200 V for 35 min. Staining and destaining were performed using eStain L1 (L00657C, Genscript, Nanjing, China).

# Purification using Ni Sepharose column

200 mL cultured bacterial solution was collected at 8000 g for 10 min and washed by ice-cold PBS twice. Cells were resuspended in 50 mL of binding buffer (50 mM Tris-HCl [pH 7.5] and 500 mM NaCl) and then sonicated (100 W, 4 s on and 6 s off intervals) on ice for 20 min using a Scientz-IID sonicator (01C1503, Scientz Biotechnology, Ningbo, China). Lysates were centrifuged at 12000 rpm for 30 min, and the supernatants were collected. The His-tagged target proteins were purified using an in-house Ni Sepharose 6FF gravity column. Namely, 1.3 g of cells from 200 mL of bacterial solution were collected, and 3 mL of Ni Sepharose 6FF resin (17531801, Cytiva, USA) (50% suspension) was used for purification. The resin was loaded into a 10 mL gravity chromatography column (7321010, Bio-Rad, USA), rinsed with 5 column volumes of double distilled water, and equilibrated with 10 column volumes of binding buffer (50 mM Tris-HCl [pH 7.5] and 500 mM NaCl). Protein supernatants were slowly added to the chromatography column. This loading process was repeated three times. The column was then washed with 10 column volumes of binding buffer and eluted with 5 column volumes elution buffers (20 mM Tris, 500 mM NaCl, and different concentrations of imidazole). The imidazole concentrations were 20 mM, 50 mM, 75 mM, 100 mM, 150 mM, 200 mM, and 300 mM, respectively.
