# Supplementary figures and images for: Expression and functional study of DNA polymerases from Psychrobacillus sp. BL-248-WT-3 and FJAT-21963
Source: Front Microbiol. 2024 Nov 20;15:1501020. doi: 10.3389/fmicb.2024.1501020 (PMC11615080; doi:10.3389/fmicb.2024.1501020)

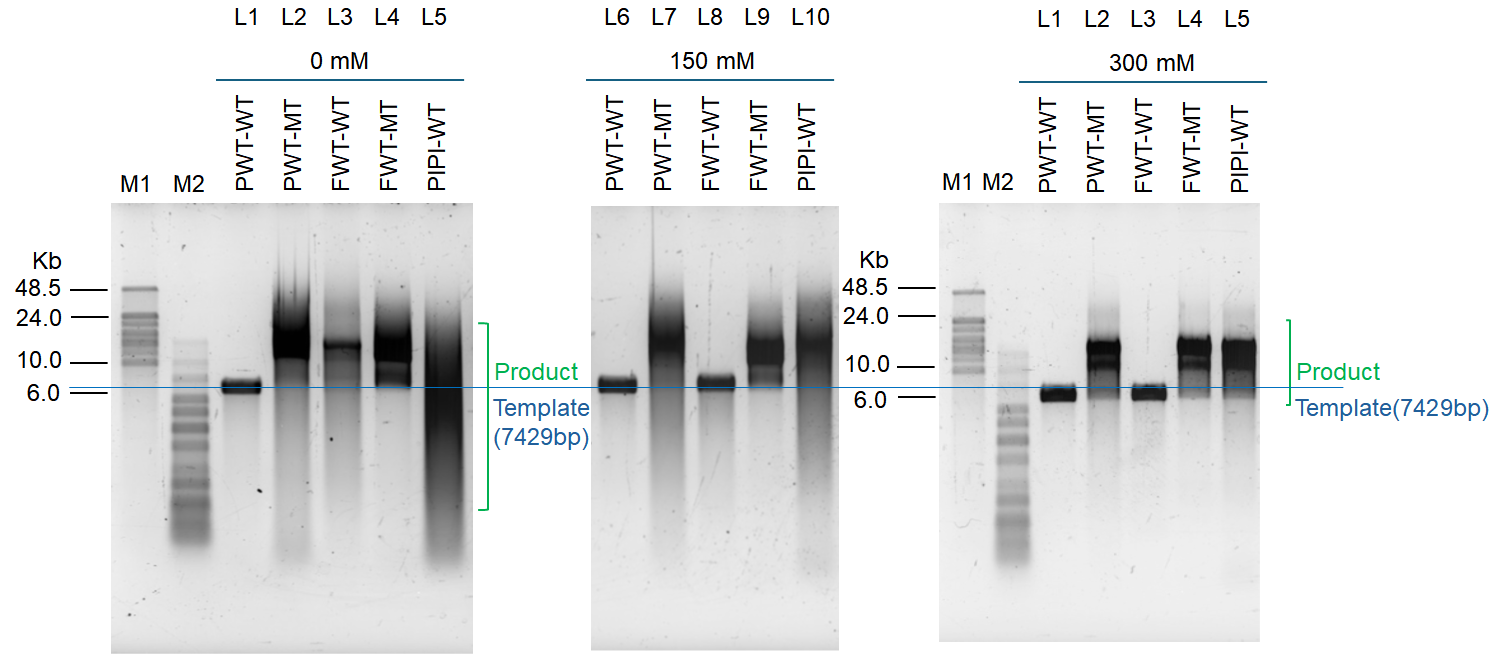

Supplement: Supplementary file 3 [file Image_1.tif]

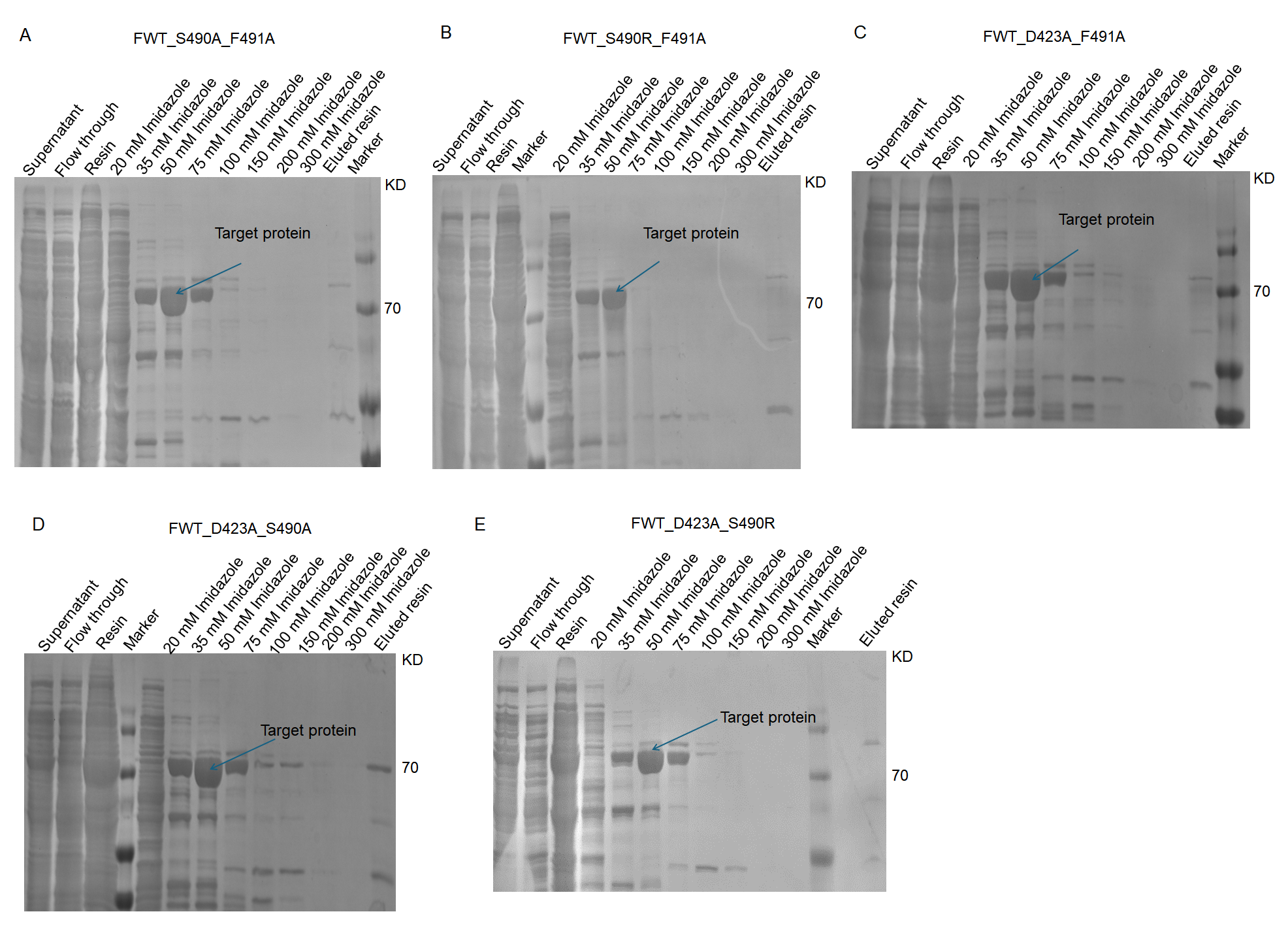

Supplement: Supplementary file 4 [file Image_2.tif]
